# Supplementary material for: A path towards single molecule vibrational strong coupling in a Fabry–Pérot microcavity
Source: Chem Sci. 2023 Jun 29;14(28):7753–61. doi: 10.1039/d3sc01411h (PMC10355109; doi:10.1039/d3sc01411h)
Supplement: SC-014-D3SC01411H-s001 [file SC-014-D3SC01411H-s001.pdf]

Electronic Supplementary Material (ESI) for Chemical Science.
This journal is © The Royal Society of Chemistry 2023

Electronic Supplementary Information for "A path towards single molecule vibrational strong coupling in a Fabry-Pérot microcavity"

Arghadip Koner[a], Matthew Du,[b] Sindhana Pannir-Sivajothi,[a] Randall H. Goldsmith,[c] and Joel Yuen-Zhou*[a]

[a]Department of Chemistry and Biochemistry, University of California San Diego, La Jolla, California 92093, USA

[b]Department of Chemistry, University of Chicago, 5735 S Ellis Ave, Chicago, Illinois 60637, USA

[c]Department of Chemistry, University of Wisconsin-Madison, Madison, Wisconsin 53706-1322, USA

*To whom correspondence should be addressed. E-mail: joelyuen@ucsd.edu

Supplementary note 1: Effective Hamiltonian

The full Hamiltonian of the optomechanical setup with the molecular vibrational mode $(b^\dagger, b)$ at frequency Ω_v and the bare cavity $(a^\dagger, a)$ at frequency ω_cav, driven by a laser modeled as a quantum harmonic oscillator $(l, l^\dagger)$ with frequency ω_L is given as

$$\begin{aligned} H_\mathrm{full} &= H_\mathrm{mol} + H_\mathrm{cav} + H_\mathrm{laser} + H_\mathrm{cm} + H_\mathrm{lc}, \\ &= \hbar\Omega_\mathrm{v} b^\dagger b + \hbar\omega_\mathrm{cav} a^\dagger a + \hbar\omega_\mathrm{L} l^\dagger l + \hbar g_0 a^\dagger a (b^\dagger + b) + \hbar J (a^\dagger + a)(l^\dagger + l), \end{aligned}$$

With the rotating wave approximation (RWA) in the laser-cavity coupling, we have

$$H_\mathrm{full}^\mathrm{RWA} = \hbar\Omega_\mathrm{v} b^\dagger b + \hbar\omega_\mathrm{cav} a^\dagger a + \hbar\omega_\mathrm{L} l^\dagger l + \hbar g_0 a^\dagger a (b^\dagger + b) + \hbar J (a^\dagger l + a l^\dagger). \tag{S1}$$

Since the cavity and the laser are linearly coupled, we can first diagonalize this part of the Hamiltonian,

$$\begin{aligned} H_\mathrm{L-C} &= \hbar\omega_\mathrm{L} l^\dagger l + \hbar\omega_\mathrm{cav} a^\dagger a + \hbar J (a^\dagger l + a l^\dagger), \\ &= \hbar\tilde{\omega}_\mathrm{L} \tilde{a}^\dagger \tilde{a} + \hbar\tilde{\omega}_\mathrm{cav} \tilde{l}^\dagger \tilde{l}, \end{aligned} \tag{S2}$$

where

$$\tilde{l} = \sin\varphi \cdot a + \cos\varphi \cdot l, \quad \tilde{\omega}_\mathrm{L} = \frac{(\omega_\mathrm{cav} + \omega_\mathrm{L}) - \sqrt{4J^2 + (\omega_\mathrm{cav} - \omega_\mathrm{L})^2}}{2},$$

$$\tilde{a} = \cos\varphi \cdot a - \sin\varphi \cdot l, \quad \tilde{\omega}_\mathrm{cav} = \frac{(\omega_\mathrm{cav} + \omega_\mathrm{L}) + \sqrt{4J^2 + (\omega_\mathrm{cav} - \omega_\mathrm{L})^2}}{2}.$$

Here, $\varphi = \frac{1}{2}\tan^{-1}\left(\frac{2J}{\omega_\mathrm{cav} - \omega_\mathrm{L}}\right)$ is the mixing angle. For small J, $\tilde{l}$ is a 'laser-like' mode and $\tilde{a}$ is a 'cavity like' mode. Rewriting $H_\mathrm{full}^\mathrm{RWA}$ in the new normal mode basis

$$H_\mathrm{full}^\mathrm{RWA} = \hbar\Omega_\mathrm{v} b^\dagger b + \hbar\tilde{\omega}_\mathrm{cav} \tilde{a}^\dagger \tilde{a} + \hbar\tilde{\omega}_\mathrm{L} \tilde{l}^\dagger \tilde{l} + \hbar g_0 \big(\cos\varphi \cdot \tilde{a} + \sin\varphi \cdot \tilde{l}\big)^\dagger \big(\cos\varphi \cdot \tilde{a} + \sin\varphi \cdot \tilde{l}\big)(b^\dagger + b).$$

For the laser being red detuned ($\omega_\mathrm{L} = \omega_\mathrm{cav} - \Delta$, $\Delta > 0$), keeping only the near resonant terms, we have

$$H_\mathrm{R} = \hbar\Omega_\mathrm{v} b^\dagger b + \hbar\tilde{\omega}_\mathrm{cav} \tilde{a}^\dagger \tilde{a} + \hbar\tilde{\omega}_\mathrm{L} \tilde{l}^\dagger \tilde{l} + \hbar g_0 \cos\varphi \sin\varphi \cdot (\tilde{l}^\dagger \tilde{a} b^\dagger + \tilde{l} \tilde{a}^\dagger b).$$

The Heisenberg equation of motion (EOM) for $(\tilde{l}^\dagger \tilde{a})$ is

$$\frac{d}{dt}(\tilde{l}^\dagger \tilde{a}) = -i(\tilde{\omega}_\mathrm{cav} - \tilde{\omega}_\mathrm{L}) \tilde{l}^\dagger \tilde{a} + i g_0 \cos\varphi \sin\varphi \cdot \left[\tilde{l} \tilde{a}^\dagger, \tilde{l}^\dagger \tilde{a}\right] \cdot b.$$

Computing the commutator

$$\left[\tilde{l} \tilde{a}^\dagger, \tilde{l}^\dagger \tilde{a}\right] = \tilde{l} \tilde{a}^\dagger \tilde{l}^\dagger \tilde{a} - \tilde{l}^\dagger \tilde{a} \tilde{l} \tilde{a}^\dagger.$$

$$\begin{aligned} \text{Using,} \quad \tilde{a}\tilde{a}^\dagger &= \tilde{a}^\dagger \tilde{a} + 1 \\ &= \tilde{l} \tilde{a}^\dagger \tilde{l}^\dagger \tilde{a} - \tilde{l}^\dagger \tilde{l} (\tilde{a}^\dagger \tilde{a} + 1) \\ &= \tilde{a}^\dagger \tilde{a} \cdot [\tilde{l}, \tilde{l}^\dagger] - \tilde{l}^\dagger \tilde{l} \\ &= \tilde{a}^\dagger \tilde{a} - \tilde{l}^\dagger \tilde{l}. \end{aligned}$$

Then,

$$\begin{aligned} \frac{d}{dt}(\tilde{l}^\dagger \tilde{a}) &= -i(\tilde{\omega}_\mathrm{cav} - \tilde{\omega}_\mathrm{L}) \tilde{l}^\dagger \tilde{a} + i g_0 \cos\varphi \sin\varphi \cdot (\tilde{a}^\dagger \tilde{a} - \tilde{l}^\dagger \tilde{l}) \cdot b, \\ &= -i(\tilde{\omega}_\mathrm{cav} - \tilde{\omega}_\mathrm{L}) \tilde{l}^\dagger \tilde{a} - i g_0 \cos\varphi \sin\varphi \cdot (\tilde{l}^\dagger \tilde{l} - \tilde{a}^\dagger \tilde{a}) \cdot b. \end{aligned} \tag{S3}$$

We will now make the mean-field approximation to linearize the equation of motion. For the three-body operators of the form $c^\dagger c b$

(where $c = \tilde{l}$ or $\tilde{a}$), we have

$$c^\dagger c b = (\underbrace{\langle c^\dagger c\rangle}_{\text{mean}} + \underbrace{c^\dagger c - \langle c^\dagger c\rangle}_{\text{Fluctuations}}) \cdot b,$$

$$= \langle c^\dagger c\rangle \cdot b + (c^\dagger c - \langle c^\dagger c\rangle) \cdot b,$$

$$\approx \langle c^\dagger c\rangle b.$$

Here we have neglected the fluctuations in $\langle c^\dagger c\rangle$. Choosing,

$$\langle \tilde{l}^\dagger \tilde{l}\rangle = \tilde{n}_{\mathrm{L}}, \langle \tilde{a}^\dagger \tilde{a}\rangle = \tilde{n}_a,$$

and plugging these back into the equation S3

$$\frac{d}{dt}(\tilde{l}^\dagger \tilde{a}) = -i(\tilde{\omega}_{\mathrm{cav}} - \tilde{\omega}_{\mathrm{L}})\tilde{l}^\dagger \tilde{a} - ig_0 \cos\varphi \sin\varphi \cdot (\tilde{n}_{\mathrm{L}} - \tilde{n}_a) \cdot b. \tag{S4}$$

Now writing down the EOM for b,

$$\frac{d}{dt} b = -i\Omega_{\mathrm{v}} b - ig_0 \cos\varphi \sin\varphi \cdot \left(\tilde{l}^\dagger \tilde{a}\right). \tag{S5}$$

To write an effective Hamiltonian, we define a composite mode for the laser-cavity subsystem $\mathscr{A}_{\mathrm{ph}} \equiv \frac{\tilde{l}^\dagger \tilde{a}}{\sqrt{(\tilde{n}_{\mathrm{L}} - \tilde{n}_a)}}$. The EOM for operators $\mathscr{A}_{\mathrm{ph}}$ and b are

$$\frac{d}{dt}\mathscr{A}_{\mathrm{ph}} = -i(\tilde{\omega}_{\mathrm{cav}} - \tilde{\omega}_{\mathrm{L}})\mathscr{A}_{\mathrm{ph}} - ig_0 \cos\varphi \sin\varphi \cdot \sqrt{(\tilde{n}_{\mathrm{L}} - \tilde{n}_a)} \cdot b,$$

$$\frac{d}{dt} b = -i\Omega_{\mathrm{v}} b - ig_0 \cos\varphi \sin\varphi \sqrt{(\tilde{n}_{\mathrm{L}} - \tilde{n}_a)} \cdot \mathscr{A}_{\mathrm{ph}}.$$

For $J \ll \Delta$, we have $\tilde{\omega}_{\mathrm{L}} \approx \tilde{\omega}_{\mathrm{cav}} - \Delta$ and $\tilde{n}_{\mathrm{L}} \approx \langle l^\dagger l\rangle \equiv n_{\mathrm{L}}$. Also

$$\cos\varphi \sin\varphi = \frac{1}{2}\sin 2\varphi$$

$$= \frac{1}{2}\sin\left(\tan^{-1}\left(\frac{2J}{\omega_{\mathrm{cav}} - \omega_{\mathrm{L}}}\right)\right)$$

$$= \frac{J}{\sqrt{\Delta^2 + 4J^2}} \approx \frac{J}{\Delta}.$$

In this limit, the EOMs transform to

$$\frac{d}{dt}\mathscr{A}_{\mathrm{ph}} = -i\Delta\mathscr{A}_{\mathrm{ph}} - ig_0\left(\frac{J}{\Delta}\right) \cdot \sqrt{(n_{\mathrm{L}} - \tilde{n}_a)} \cdot b,$$

$$\frac{d}{dt} b = -i\Omega_{\mathrm{v}} b - ig_0\left(\frac{J}{\Delta}\right)\sqrt{(n_{\mathrm{L}} - \tilde{n}_a)} \cdot \mathscr{A}_{\mathrm{ph}}.$$

Now using the fact that $n_{\mathrm{L}} \gg \tilde{n}_a$, we have

$$\frac{d}{dt}\mathscr{A}_{\mathrm{ph}} = -i\Delta\mathscr{A}_{\mathrm{ph}} - ig_0\left(\frac{J}{\Delta}\right) \cdot \sqrt{n_{\mathrm{L}}}\sqrt{1 - \frac{\tilde{n}_a}{n_{\mathrm{L}}}} \cdot b,$$

$$\approx -i\Delta\mathscr{A}_{\mathrm{ph}} - ig_0\left(\frac{J}{\Delta}\right)\sqrt{n_{\mathrm{L}}} \cdot b,$$

$$\frac{d}{dt} b = -i\Omega_{\mathrm{v}} b - ig_0\left(\frac{J}{\Delta}\right)\sqrt{n_{\mathrm{L}}} \cdot \mathscr{A}_{\mathrm{ph}}.$$

These EOMs look like two coupled oscillators with coupling constant $g_0\left(\frac{J}{\Delta}\right)\sqrt{n_{\mathrm{L}}}$. Thus, we can write an effective Hamiltonian for this system as

$$H_{\rm eff} = \hbar\Delta\mathscr{A}^\dagger_{\rm ph}\mathscr{A}_{\rm ph} + \hbar\Omega_{\rm v} b^\dagger b + \hbar g_0\left(\frac{J}{\Delta}\right)\sqrt{n_{\rm L}}\left(\mathscr{A}^\dagger_{\rm ph} b + \mathscr{A}_{\rm ph} b^\dagger\right). \tag{S6}$$

We note that for $J \ll \Delta$ and $n_{\rm L} \gg \tilde{n}_a$, $\{\mathscr{A}_{\rm ph}, \mathscr{A}^\dagger_{\rm ph}\}$ satisfy bosonic commutation relations,

$$\begin{aligned}
[\mathscr{A}_{\rm ph}, \mathscr{A}^\dagger_{\rm ph}] &= \frac{\tilde{l}^\dagger\tilde{a}\tilde{l}\tilde{a}^\dagger - \tilde{l}\tilde{a}^\dagger\tilde{l}^\dagger\tilde{a}}{(\tilde{n}_{\rm L} - \tilde{n}_a)}, \\
&= \frac{\tilde{l}^\dagger\tilde{l} - \tilde{a}^\dagger\tilde{a}}{(\tilde{n}_{\rm L} - \tilde{n}_a)}, \\
&\approx \mathbf{I}.
\end{aligned}$$

Supplementary note 2: Decay rate for the composite boson

The full Hamiltonian in the RWA with the decay of the cavity and the vibrational mode as κ and γ (we are assuming that the laser mode has no incohorent decay) is given as

$$H = \hbar\left(\Omega_{\rm v} - i\frac{\gamma}{2}\right)b^\dagger b + \hbar\left(\omega_{\rm cav} - i\frac{\kappa}{2}\right)a^\dagger a + \hbar\omega_{\rm L} l^\dagger l + \hbar g_0 a^\dagger a(b^\dagger + b) + \hbar J(a^\dagger l + a l^\dagger).$$

Diagonalizing the cavity-laser subsystem

$$H_{\rm L-C} = \hbar\left(\omega_{\rm cav} - i\frac{\kappa}{2}\right)a^\dagger a + \hbar\omega_{\rm L} l^\dagger l + \hbar J(a^\dagger l + a l^\dagger),$$

the normal mode frequencies are

$$\begin{aligned}
\tilde{\omega}_{\rm L} &= \frac{\omega_{\rm L} + (\omega_{\rm cav} - i\kappa/2) - \sqrt{4J^2 + [(\omega_{\rm cav} - i\kappa/2) - \omega_{\rm L}]^2}}{2}, \\
\tilde{\omega}_{\rm cav} &= \frac{\omega_{\rm L} + (\omega_{\rm cav} - i\kappa/2) + \sqrt{4J^2 + [(\omega_{\rm cav} - i\kappa/2) - \omega_{\rm L}]^2}}{2}.
\end{aligned}$$

Now, we need the decay for the composite bosons. Let's consider the Hamiltonian

$$H_0 = \hbar\tilde{\omega}_{\rm L}\tilde{l}^\dagger\tilde{l} + \hbar\tilde{\omega}_{\rm cav}\tilde{a}^\dagger\tilde{a}.$$

In the Heisenberg picture

$$\tilde{l}(t) = \tilde{l}e^{-i\tilde{\omega}_{\rm L}t}, \quad \tilde{a}(t) = \tilde{a}e^{-i\tilde{\omega}_{\rm cav}t}.$$

The Heisenberg EOM for $(\tilde{l}^\dagger\tilde{a})$ is

$$\begin{aligned}
\frac{d}{dt}(\tilde{l}^\dagger\tilde{a}) &= \frac{i}{\hbar}[H_0, \tilde{l}^\dagger\tilde{a}] \\
&= -i(\tilde{\omega}_{\rm cav} - \tilde{\omega}_{\rm L})\tilde{l}^\dagger\tilde{a}.
\end{aligned}$$

Thus

$$\tilde{l}^\dagger\tilde{a}(t) = \tilde{l}^\dagger\tilde{a}e^{-i(\tilde{\omega}_{\rm cav} - \tilde{\omega}_{\rm L})t},$$

where

$$\begin{aligned}\tilde{\omega}_{\mathrm{cav}} - \tilde{\omega}_{\mathrm{L}} =& \frac{(\omega_{\mathrm{cav}} - i\kappa/2) + \omega_{\mathrm{L}} + \sqrt{4J^2 + [(\omega_{\mathrm{cav}} - i\kappa/2) - \omega_{\mathrm{L}}]^2}}{2} - \\ & \frac{(\omega_{\mathrm{cav}} - i\kappa/2) + \omega_{\mathrm{L}} - \sqrt{4J^2 + [(\omega_{\mathrm{cav}} - i\kappa/2) - \omega_{\mathrm{L}}]^2}}{2}, \\ =& \sqrt{4J^2 + [(\omega_{\mathrm{cav}} - i\kappa/2) - \omega_{\mathrm{L}}]^2}, \\ \approx& (\omega_{\mathrm{cav}} - \omega_{\mathrm{L}}) - i\kappa/2,\end{aligned}$$

for $J \ll (\omega_{\mathrm{cav}} - \omega_{\mathrm{L}})$.

Thus, we show that the incoherent decay rate for the composite boson is the same as that of the cavity decay assuming that the laser mode has no incoherent decay. The full Hamiltonian in the normal mode basis is then given as

$$H = \hbar\left(\Omega_{\mathrm{v}} - i\frac{\gamma}{2}\right)b^\dagger b + \hbar\left((\omega_{\mathrm{cav}} - \omega_{\mathrm{L}}) - i\frac{\kappa}{2}\right)\mathscr{A}^\dagger_{\mathrm{ph}}\mathscr{A}_{\mathrm{ph}} + \hbar g_0\left(\frac{J}{\Delta}\right)\sqrt{n_{\mathrm{L}}}\left(\mathscr{A}^\dagger_{\mathrm{ph}} b + \mathscr{A}_{\mathrm{ph}} b^\dagger\right).$$
